# Compound Heat Transfer Augmentation of a Shell-and-Coil Ice Storage Unit with Metal-Oxide Nano Additives and Connecting Plates

**DOI:** 10.3390/nano12061010

**Published:** 2022-03-18

**Authors:** Farhad Afsharpanah, Seyed Soheil Mousavi Ajarostaghi, Farzam Akbarzadeh Hamedani, Mohsen Saffari Pour

**Affiliations:** 1 Mechanical Engineering Department, Babol Noshirvani University of Technology, Babol 47148, Iran; f.afsharpanah@gmail.com (F.A.); soheilmousavi67@gmail.com (S.S.M.A.); 2 Mechanical Engineering Department, Chabahar Maritime and Marine University, Chabahar 99717, Iran; farzam.akbarzadeh@gmail.com; 3 Mechanical Engineering Department, Faculty of Engineering, Shahid Bahonar University of Kerman, Kerman 76169, Iran

**Keywords:** CFD analysis, numerical simulation, PCM, nanoparticles, ice storage, solidification, thermal energy storage, phase change process, heat transfer enhancement, heat exchanger

## Abstract

Due to the high enthalpy of fusion in water, ice storage systems are known as one of the best cold thermal energy storage systems. The phase change material used in these systems is water, thus it is inexpensive, accessible, and completely eco-friendly. However, despite the numerous advantages of these systems, the phase change process in them is time-consuming and this leads to difficulties in their practical application. To solve this problem, the addition of nanomaterials can be helpful. This study aims to investigate the compound heat transfer enhancement of a cylindrical-shaped unit equipped with double helically coiled coolant tubes using connecting plates and nano additives as heat transfer augmentation methods. Complex three-dimensional numerical simulations are carried out here to assess the best heat exchanger material as well as the impact of various nanoparticle types, including alumina, copper oxide, and titania, and their concentrations in the PCM side of the ice storage unit. The influence of these parameters is discussed on the charging rate and the temperature evolution factor in these systems. The results suggest that using nano additives, as well as the connecting plates, together is a promising way to enhance the solidification rate by up to 29.9%.

## 1. Introduction

The world population and energy demand are rising every day. When the energy demand and production are not aligned, the production units should be designed for the peak energy demand. This means a higher number of facilities and more-priced equipment are needed to meet energy demand, on the other hand, when the energy demand is distributed throughout the day, supplying energy is easier [1]. Distribution of the energy demand throughout the day is known as load-leveling. Load-leveling can be obtained by encouragement policies that are usually applied by some governments to modify the consumption pattern in society. Setting different prices for electricity in the low-load or peak-demand times is one of these policies. These policies encourage people to change their consumption behavior and to use energy storage systems. There are various types of energy storage systems, one of the most important of which is thermal energy storage (TES). Huggins [1] states that 10.3% of the total energy demand belongs to the energy consumption in commercial and residential buildings. The heating and cooling of these spaces are probably one of the major parts of this energy demand. TES systems facilitate load-leveling for these applications.

Ice storage systems are one of the cold latent TES (CLTES) systems that mostly rely on the significant enthalpy of fusion of water. This type of CLTES system can shift the cooling load to achieve a more balanced demand pattern during the day. In these systems, the energy is stored by freezing the water using a cold heat transfer fluid (HTF) and the energy release is obtained by melting the solidified water. The obtained cold energy can be used for cooling different spaces, devices, etc. Aside from the numerous advantages of ice storage systems, the low charging and discharging speeds act as impediments to the generalization of these systems. Some of the techniques that are probably effective to solve these problems are the conventional heat transfer augmentation methods such as twisted tape insertion on the HTF side, using nano additives, metal foams [2], special HTF tube types, and fins. 

Nanoparticles (NPs) are particles of matter with sizes varying between 1 and 100 nm. These particles have unique magnetic, chemical, electrical, and, optical characteristics. NPs may have different shapes such as spheres, cubes, and prisms. They have a wide range of potential and actual applications, in various sciences such as biology, materials science, chemistry, engineering, and physics. They also have extensive applications in transportation, medicine, environmental science, and energy [3]. Research studies have shown that the addition of NPs with high thermal conductivity can improve the thermal conductivity of fluids [4,5,6]. Also, some studies have revealed that the addition of these NPs can enhance the phase change rate in phase change materials (PCMs) [7,8,9].

Al-Madhhachi [10] worked on the optimization of a solar portable refrigerator unit for vaccines. Their cooling unit worked with solar panels and it was utilized with heat sinks. They studied the efficiency and heat dissipation of the unit. They stated that this solar system is more affordable than the other similar systems. Also, according to their results, the highest obtainable surface efficiency in this system was 78%. In the research conducted by Najim et al. [11], circular fin arrays with non-uniform dimensions were employed to improve the melting rate in a vertical triple-tube (TT) PCM unit. They studied the impact of the distribution and characteristics of these fins on the enhancement of this process. They managed to obtain faster charging rates by up to 10.4%, by adjusting the non-uniform fin dimensions. Alnakeeb et al. [12] worked on the optimization of an inner-flat tube double-tube PCM unit by creating geometric alternations in the tube and the eccentricity. The tested range of aspect ratios for this type of tube was between 0.5 and 1, while the eccentricity ranged between 0 and 0.75. They discovered that with the eccentric circular tube with an eccentricity of 0.75, the melting rate can be elevated by up to 218% higher than the concentric mode. They also deduced that lower aspect ratios for this type of inner tube results in faster melting rates.

Leng et al. [13] experimentally investigated the melting and solidification process of two shell-and-tube (SAT) PCM units with one or four inner HTF tubes. They kept the amount of the PCM and the HTF flow rate constant for both of the models. They revealed that the model with four inner tubes improves the theoretical efficiency of the unit from 75.9% for the single-tube model to 90.3%. They revealed that even though the four-tube model creates a higher friction loss, its efficiency and heat-energy ratio are significantly better. Li et al. [14] evaluated the effects of distribution, number, and temperature of HTF tubes on the improvement of the solidification process of RT35 wax in a multi-tube TES unit. They examined the changes in the liquid fraction and energy release rate during the process. Based on their obtained results, they recommended placing the HTF tubes near the middle area of the unit. They also discovered that raising the distance between the HTF tubes leads to detrimental results. It was found that increasing the HTF tube temperature from 5 to 10 and 15 °C can improve the solidification rate by up to 7 and 16%. Shahsavar et al. [15] conducted some numerical simulations to assess the influence of the sinusoidal fins on the enhancement of charging and discharging processes of a TES. They examined a vertical double pipe, in which the inner and outer pipes were occupied with the PCM and HTF flow. They examined various wavelengths and wave amplitudes to recognize the best wavy fin profile. According to their results, the fin wavelength and amplitude of 1 and 2 mm were recommended, respectively. Compared to when smooth fins were used, these optimized fins could raise the solidification and melting rates by up to 4 and 7%, respectively.

Mahdavi et al. [16] worked on the simultaneous melting and solidification process in a multi-tube HX, in which paraffin was employed as the PCM. They used a pattern of several hot and cold tubes to observe the system in this condition. They revealed that the position of these tubes determines the steady-state liquid fraction of the unit. They also tested the impact of NPs and stated that the use of NPs in this condition is not recommended. In their study, it was also suggested that the fins are not very influential in this condition, with any influence limited to one of the tube arrangements. Li et al. [17] utilized the Galerkin method for the examination of the phase change phenomenon of PCM, in which nanomaterial additives were dispersed. They completely neglected the influence of gravity, velocity, and natural convection for their examination, since the influence of natural convection on the freezing process is not considerable. Therefore, the governing equation was significantly simplified. On the other hand, they employed an adaptive grid for the near fin area, to enhance the solution accuracy. Their study focused on investigating different NP shapes. They discovered that at maximum, NPs lead to an 11% faster freezing rate. They recommended the platelet shape NPs due to their higher shape factor. Mundra and Pardeshi [18] used both computational and experimental techniques to analyze the performance of a horizontal TES unit. To improve the response time of the unit they used graphene nanoplatelets. They tested NP mass fractions of up to 4%. Based on their results, the addition of NPs improved the overall phase change rate and enhanced the system performance. Zhang et al. [19] carried out a two-dimensional (2D) numerical analysis to evaluate the thermal performance of a PCM-embedded glazing window. They tested zinc oxide, titania, and alumina NPs for the enhancement of the thermal properties of the PCM (Paraffin). They showed that the addition of alumina NPs in the paraffin raises the glass temperature. They also stated that even though due to the low ambient temperature in winter PCM remains in the solid phase, the existence of NPs still rises the inner surface temperature. 

In the study of Afsharpanah et al. [20], efforts were made to optimize two geometric variables regarding the HX of a CLTES unit. In their investigation, they evaluated the influence of these variables on the charging rate of the unit. They solely focused on these two geometric variables, and no flow parameters were studied. They also ignored the flow inside the tubes. In another investigation [21], they expanded their previous research to assess more variables concerning the HX, fins, and coolant flow. They introduced connecting plates for improving the heat transfer. They assessed the impact of these variables that were related to the HX, connecting plates, and coolant flow, on the charging rate of the unit. They limited the simulation time to four hours, mainly due to the computational cost. They revealed that the best HX, had a dual helical coil design with three helix having a pitch length of 54 mm, helix distance of 45 mm, tube inner diameter of 16.55 mm, tube thickness of 1.25 mm, and a connecting plate length of 162 mm. Furthermore, the maximum freezing rate was obtained with a coolant temperature of −10 °C and a Reynolds number of 2000. 

As was explained, even though ice storage systems have lots of applications, their thermal conductivity is low and this creates problems for the generalization of these devices. The literature review suggested that the researchers are mostly focusing on the other types of PCM since their simulation is more simple (significantly lower simulation time, and in many cases identical values for thermophysical properties in solid and liquid phases). Moreover, the majority of the performed numerical studies in the field of PCM are two-dimensional (2D) and over-simplified. This paper presents a complex three-dimensional (3D) analysis for the enhancement of the freezing rate in a cylindrical-shaped ice storage container that has been enhanced by the dispersion of metal oxide nanomaterials with high thermal conductivities. The boundary conditions used for this study are significantly closer to reality, and commercial materials are selected to present a more realistic evaluation. At first, three commercial materials are tested to select the best material for the HX tubes and fins, then a compound enhancement technique is used to improve the charging rate. The utilized method includes nano additives and connecting plates. Three different nanomaterial additives and volume fractions are tested to assess the best one for this application. The influence of these parameters is studied on the liquid fraction and the temperature evolution factor. 

## 2. Computational Simulation

In this section, the steps taken for obtaining the computational results will be discussed. 

### 2.1. Problem Description

**Figure 1:**
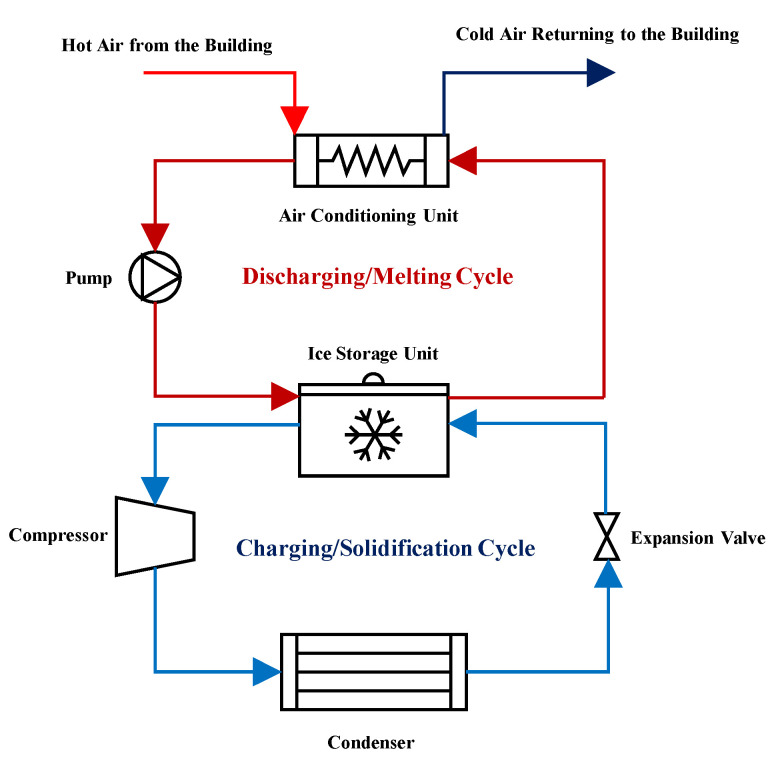
The charging and discharging processes of an ice storage unit.

To better understand the application of the ice storage units, their charging, and discharging cycles have been depicted in Figure 1. As shown in this figure, for charging these devices, conventional compression refrigeration cycles are used at the off-peak hours, while for discharging them, they can be coupled with the air-conditioning units of the building at peak hours, and provide cooling without putting pressure on the power network system.

**Figure 2:**
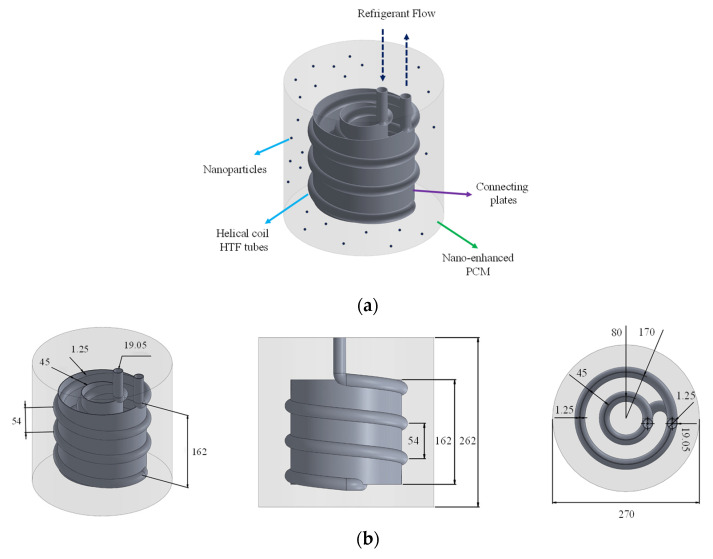
The examined CLTES unit: (**a**) the schematic; (**b**) geometrical specifications.

This research tries to examine the influence of the HX materials as well as the compound improvement of the solidification process by the means of nano additives and connecting plates on the charging process of an ice storage unit. The schematic of the problem is given in Figure 2. As illustrated, the system consists of four parts:

1. The coolant helical coils carry the coolant flow to cool down the storage;
2. The water in the container acts as the PCM and freezes as the heat in the container is absorbed by the coolant tubes;
3. The plates are used as fins to distribute the coldness of the tubes more uniformly in the container;
4. Nanomaterial additives are added to the PCM to thermally enhance it and improve its thermal conductivity.

The optimized geometry of Afsharpanah et al. [21] is used as the basis of this study to further improve the process through other methods, and also to present a more precise set of results by considering a much longer simulation time. The geometric specifications have been graphically indicated in Figure 2b. It is noteworthy that for comparison, some simulations are also conducted without the plates. Furthermore, the temperature and Reynolds numbers of −10 °C and 2000 are chosen for the refrigerant flow. Water is selected as the PCM, and the refrigerant flow is a mixture of water and ethylene glycol (7:3 in vol.). Stainless steel AISI 304, aluminum alloy 6061, and copper alloy C11000 are tested for the HX (both helical coil and plates). Also, aluminum-oxide, copper (II) oxide, and titanium dioxide NP materials are utilized. The thermophysical properties of the materials are provided in Table 1.

Aluminum oxide (Al_2_O_3_) NPs with an average diameter of 50 nm, are in the form of a white powder that can be dispersed within the PCM. Copper (II) oxide (CuO) NPs are in the form of a black powder and have an average diameter of 20 nm. Similar to the aluminum oxide NPs, titanium dioxide (TiO_2_) NPs are in the form of a white powder, however, their average diameter is 30 nm. Despite the difference in the crystalline structure of these nanoparticles, the morphological shape of all of them is semi-spherical. As can be observed in Table 1, the thermal conductivity of these NPs is considerably higher than water, thus, dispersing them within the PCM can considerably improve its thermophysical properties. 

Overall, eight cases were defined to assess the influence of three parameters, including the HX material, NP type, and NP concentration. The cases are presented in detail in Table 2. It is worth noting that all of these cases have a similar geometry, which has been illustrated in Figure 2b. For comparative purposes, a case zero was also created, in which the plates were removed and no NPs were used.

The assumptions of this study are listed below:

1. The process is transient;
2. The flow within the entire system is incompressible and laminar;
3. Various thermophysical values are applied for ice and water;
4. The volume change throughout the process is ignored;
5. The Boussinesq approximation is utilized to contemplate the results of natural convection;
6. The NPs are homogeneously dispersed within the PCM using an ultrasonic homogenizer;
7. The insulation layers over the storage are thick enough to impose a heat flux of zero.

### 2.2. Governing Equations, Solution Condition, and Method

The thermophysical properties of the nano-enhanced PCM (NEPCM) were obtained based on the properties of the base fluid and NPs. For this purpose the following equations were utilized [6,7]:

(1) $$\mu_{NEPCM}=\frac{\mu_{b}}{{\left(1-\phi \right)}^{2.5}}$$

(2) $$\rho_{NEPCM}=(1-\phi )\rho_{b}+\phi \rho_{np}$$

(3) $$\beta_{NEPCM}=(1-\phi )\beta_{b}+\phi \beta_{np}$$

(4) $$\frac{k_{NEPCM}}{k_{b}}=\frac{\left(k_{np}+2k_{b}\right)-2\phi \left(k_{b}-k_{np}\right)}{\left(k_{np}+2k_{b}\right)+\phi \left(k_{b}-k_{np}\right)}$$

(5) $${\left(\rho c_{p}\right)}_{NEPCM}=(1-\phi ){\left(\rho c_{p}\right)}_{b}+\phi {\left(\rho c_{p}\right)}_{np}$$

The enthalpy of fusion for the NEPCM was calculated using [7]:

(6) $${\left(\rho h_{sf}\right)}_{NEPCM}=(1-\varphi ){\left(\rho h_{sf}\right)}_{b}$$

The continuity:

(7) $$\nabla \cdot \vec{v}=0$$

Momentum:

(8) $$\frac{\partial v}{\partial t}+\vec{v}\cdot \nabla \vec{v}=\frac{1}{\rho }\left(-\nabla \rho +\mu \nabla^{2}\vec{v}+\rho \beta \left(T-T_{ref}\right)\right)-\frac{{\left(1-\lambda \right)}^{2}}{\lambda^{3}}C_{mush}\vec{v}$$

and Energy equation:

(9) $$\frac{\partial h_{sens}}{\partial t}+\frac{\partial h_{lat}}{\partial t}+\nabla \cdot \left(\vec{v}h_{sens}\right)=\nabla \cdot \left(\frac{k}{\rho c_{p}}\nabla h_{sens}\right)$$

where:

(10) $$h_{tot}=h_{sens}+h_{lat}$$

The freezing progress was modeled with the aid of the enthalpy-porosity technique.

(11) $$h_{tot}=h_{sens}+h_{lat}$$

(12) $$h_{lat}=\sum_{i=1}^{n}\lambda_{i}h_{sf}$$

In the above equations, *λ* designates the liquid fraction, which ranges in 0 ≤ *λ* ≤ 1. This value determines the cell state from the mushy (0< *λ*< 1), solid (λ = 0), and liquid (*λ* = 1) options. The mushy cells act similar to a porous area, the porosity of which is equal to this value. This variable is written as [23]:

(13) $$\lambda =\left\{\begin{array}{l} \frac{h_{lat}}{h_{sf}}=0\;if\;T\leq T_{solidus}\\ \frac{h_{lat}}{h_{sf}}=1\;if\;T\geq T_{\mathrm{liquidus}}\\ \frac{h_{lat}}{h_{sf}}=\frac{T-T_{solidus}}{T_{\mathrm{liquidus}}-T_{solidus}}\;if\;T_{solidus}<T<T_{\mathrm{liquidus}} \end{array}\right\}$$

where C_mush_ with a value of 10^5^ kg/s·m^3^ shows the constant of the mushy zone [24]. The Boussinesq approximation accounts for the impact of natural convection:

(14) $$\rho =\rho_{0}{\left[\beta \left(T-T_{liquidus}\right)+1\right]}^{-1}$$

Also, to evaluate the progression of the temperature alternations in the unit, a dimensionless parameter, namely, the temperature evaluation factor (TEF) was defined:

(15) $$\theta =\frac{T-T_{init}}{T_{cool,i}-T_{init}}$$

A TEF of zero shows that a cell is at the initial temperature, while a value of one for this variable reveals that the cell has reached the inlet temperature of the coolant. Values in the range between zero and one indicate the status of the cell in this progress. 

The governing equations were solved considering the following conditions:

A temperature and velocity component field of +1 °C and 0 m/s was selected as the beginning state of the unit. Inlet and outlet of the coolant flow were defined to have velocity inlet and pressure outlet boundaries, respectively. The first one was set with a velocity of 0.6164 m/s, and a temperature of −10 °C, while the latter one was set with a value of zero Pa as the gauge pressure. 

This paper uses a numerical method for simulating the process. For this purpose, by assuming that the NPs are homogeneously dispersed within the PCM (by using an ultrasonic homogenizer), at first, the homogenous model [7] (Equations (1)–(6)) is employed to calculate the thermophysical properties of the NEPCM, based on thermophysical properties of the base PCM and NPs in the Engineering Equation Solver 10.561. After calculating the thermophysical properties of the NEPCM with different nanomaterials and various concentrations, these thermophysical properties are inserted in ANSYS Fluent 21R2 software, which is utilized for solving the heat and mass transfer governing equations by considering the solution conditions. This software uses the computational fluid dynamic (CFD) method with a finite volume approach. In this technique, the governing equations are solved for each time step, and the termination criteria for each time step is reaching residuals equal to 10^−6^ for the energy and 10^−4^ for velocity components and continuity. The simulations continue until the liquid fraction reaches the value of 0.05, which is considered complete solidification. 

### 2.3. Validation and Sensitivity Analyses

**Figure 3:**
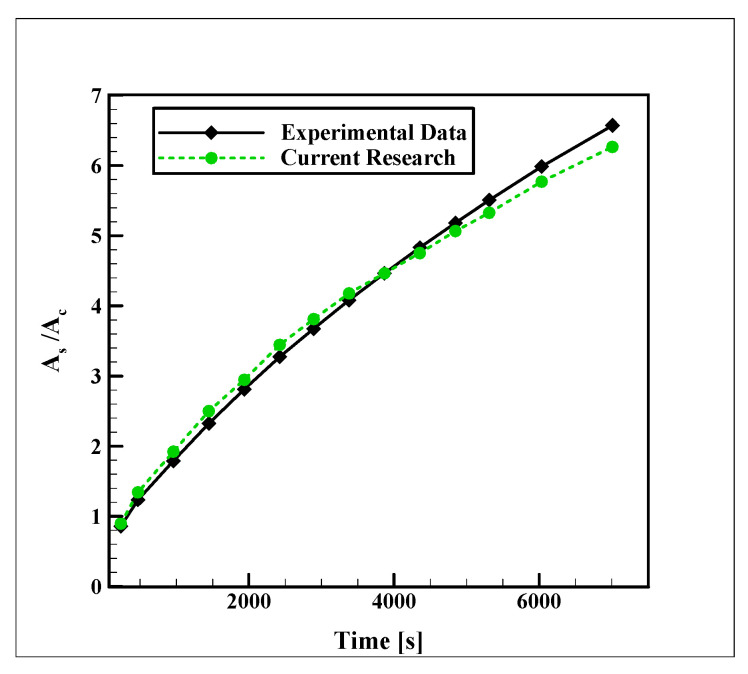
Validation of the current numerical simulations with the experimental data of Sasaguchi et al. [23].

Due to the lack of experimental data on devices similar to those studied in this research, the numerical simulations are validated with the study of Sasaguchi et al. [25], in which they examined the water freezing process in a rectangular type SAT unit (with the length of 177.8 mm and the width of 101.6), containing two HTF pipes with a diameter of 25.4 mm. The distance between the center points of these two pipes was 50.8 mm. Also, the distance of the center point of the top pipe from the top storage wall was 63.5 mm, and the bottom pipe had the same distance from the bottom storage wall. The pipes were carrying coolant with a temperature of −10 °C. Figure 3 illustrates a comparison between the current computational simulations and their experimental results for the ratio of the frozen area to the cross-section area of the tubes. The maximum deviation of 8.1% between these data reveals the good performance of the computational code for predicting the process. 

**Figure 4:**
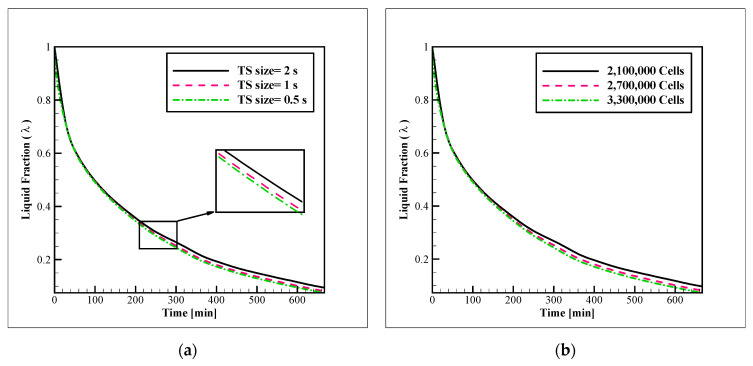
Sensitivity of the simulations to: (**a**) The time step size; (**b**) The number of cells.

**Figure 5:**
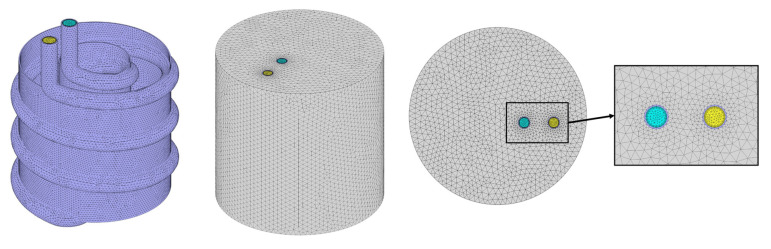
The selected mesh network for simulations.

The sensitivity of the numerical model to the size of the time-step (TS) and mesh network was evaluated for Case 2. The mesh networks with 2,100,000, 2,700,000, 3,300,000 cells were tested while the examination of the TS size was conducted with the TS sizes of 2, 1, and 0.5 s. The results are illustrated in Figure 4a, b. Based on the close compatibility of the results with 2,500,000 cells and TS size of 1 s, with the obtained results with their finer counterparts, the refinement was stopped and these conditions were chosen for the simulations. The mesh network, which was employed for the simulations, is displayed in Figure 5. As shown in this figure, adaptive grids with finer mesh cells were utilized on the regions near the coolant tubes and plates. This mesh network was generated with the element sizes of 7, 2, and 2 mm for the PCM, coolant, and tube zones, respectively. The coolant zone was covered with five layers of boundary layer mesh with a first-layer thickness of 0.2 mm and a growth rate of 1.2. Regarding the grid controlling strategies, the study of Sun et al. [26] was useful. 

## 3. Results

In this section, the influence of several variables is studied on the alternations of the liquid fraction and temperature evolution factor within the unit. At first, three materials, including aluminum alloy 6061, copper alloy C11000, and stainless steel AISI 304 are tested for the coils and plates. After selecting the superior material, the simulations are continued in the presence of various types of nanoparticles, namely, Al_2_O_3_, CuO, and TiO_2_ at the NP concentration of 3%, to assess the best NPs for the unit. In the final stage, several concentrations of the best NP considering the freezing rate are examined. These investigations are performed in three separate sections. It is worth noting that the complete freezing in all of the simulations is considered to be the liquid fraction of 0.05, and this is the termination criterion for stopping the simulation for each case. 

### 3.1. Heat Exchanger Material

**Figure 6:**
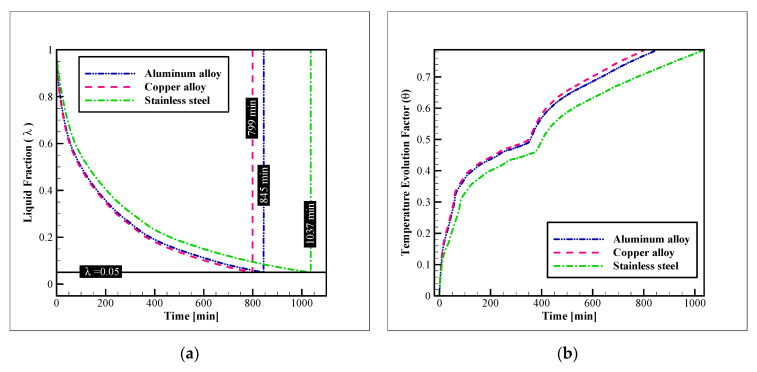
The influence of the HX material on: (**a**) The liquid fraction reduction; and (**b**) TEF advancement.

In this section, the impact of selecting several commercial alloys as the HX material is studied. The following figure (Figure 6) shows this influence on liquid fraction reduction and the progression of the temperature evolution factor (TEF). As designated in the figure, copper alloy presents the best thermal response by providing a complete solidification in less than 799 min. Aluminum alloy has the second rank, by presenting a complete freezing time of 845 min. The stainless steel alloy exhibits the worst performance in this regard. By considering these times, it is clear that the freezing time of the copper alloy is 5.44%, and 22.95% better than aluminum and stainless steel alloys. The same pattern is observed for the TEF diagram, as copper alloy shows the faster advancement in the temperature change towards the coldest possible state of the system (coolant inlet temperature). The reason for this difference is the higher thermal conductivity of copper alloys compared to the other two options. The other important point to keep in mind is the density of the material. Comparing the density of the copper and aluminum alloys shows that the density of the copper alloy is virtually 70% higher than that of the aluminum alloy (Table 1). Therefore, considering the volume of the tubes and the connecting plates are the same, the lower density of the aluminum alloy leads to a much lighter HX configuration compared to copper alloy. On the other hand, the thermal performance with these materials is not much different. Thus, the selection of aluminum or copper alloy for the HX depends on the application of the unit, and whether the weight is an important parameter for that application or not. Also, the aluminum alloy is cheaper, and it is another fact to consider while selecting one of these two materials. 

**Figure 7:**
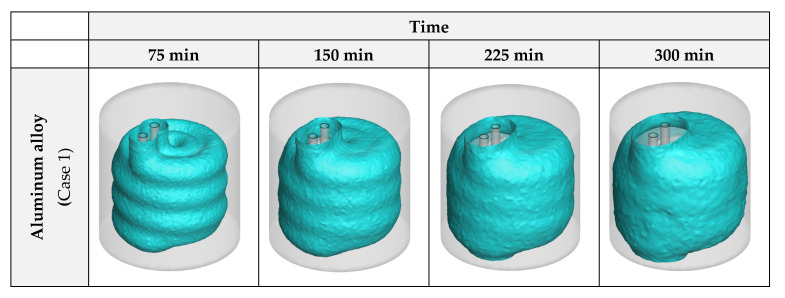

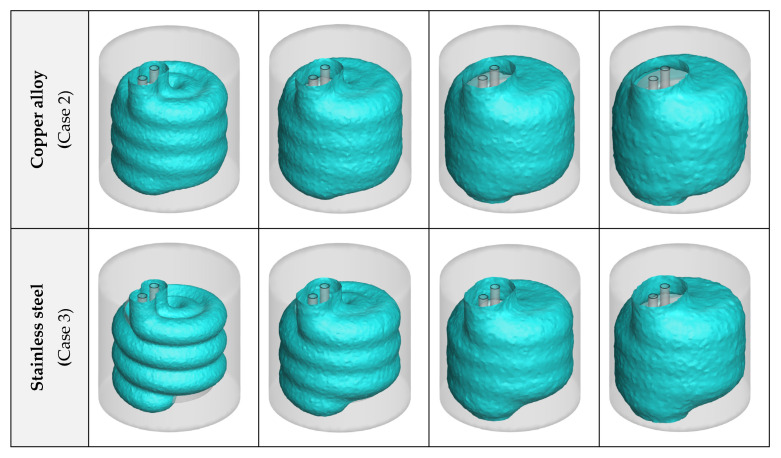
The 3D zero-liquid fraction iso-surfaces with various HX materials.

To properly compare the influence of the HX material on the advancement of the progress, 3D iso-surfaces of zero liquid fraction (λ = 0) were generated as an indication of the formed ice layers in the unit. These images can be found in Figure 7. As can be comprehended from the figure, while aluminum and copper alloys exhibit similar results, stainless steel presents an unsatisfactory performance compared to them. 

### 3.2. Nanoparticle Material

**Figure 8:**
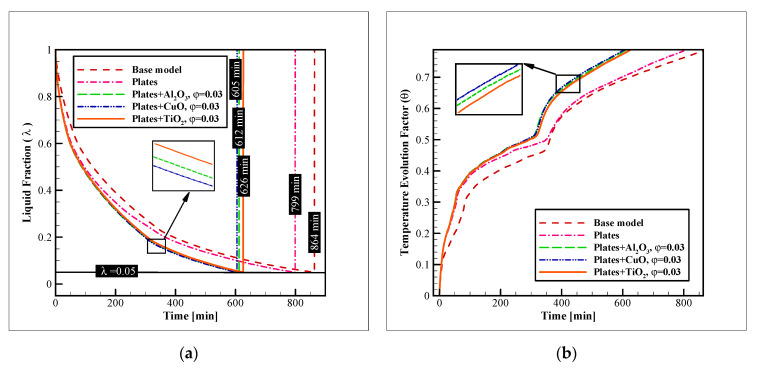
The impact of the NP material and presence of plates on: (**a**) The liquid fraction reduction; and (**b**) TEF advancement.

To further improve the process, a combination of connecting plates and nano additives was chosen in this study. In the first step, the dispersion of several types of NPs was tested. These NPs were alumina, copper oxide, and titania, and in this section, their concentration was fixed at 3% (in volume). The enhancement on the liquid fraction reduction and TEF advancement with these augmentation techniques were compared to the base model, without the connecting plates or NPs. Figure 8 shows the results of this analysis. As illustrated, the base model had the worst performance with a complete freezing time, which is as long as 864 min. The addition of plates improved the time to 799 min (7.52% of improvement). The dispersion of titania, alumina, and copper oxide NPs enhanced the freezing time even further to 626, 612, and 605 min (27.54, 29.16, and 29.97% of enhancement compared to the base model, and 21.65, 23.40, and 24.28% compared to the model with the pure PCM and connecting plates). Observing these values reveals that while the dispersion of NPs can significantly augment the freezing rate, the NP type is not very influential. The difference of the complete freezing time with copper oxide NPs and the alumina and titania NPs is only 1.1, and 3.3%, respectively. Thus, for selecting the material of the NPs for dispersion in PCMs, mostly their price and availability should be considered, since their influence on the process is not much dependent on the material, and the addition of any type of these NPs can considerably enhance the process. This point is more evident in the 3D iso-surface images.

**Figure 9:**
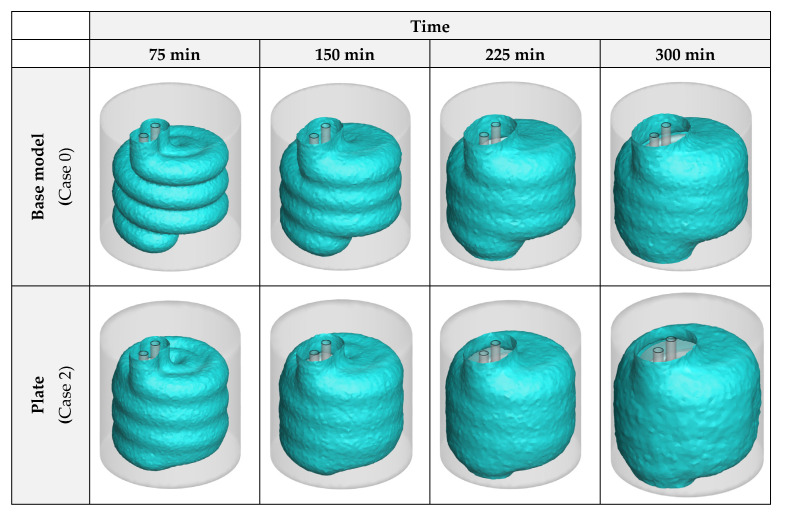

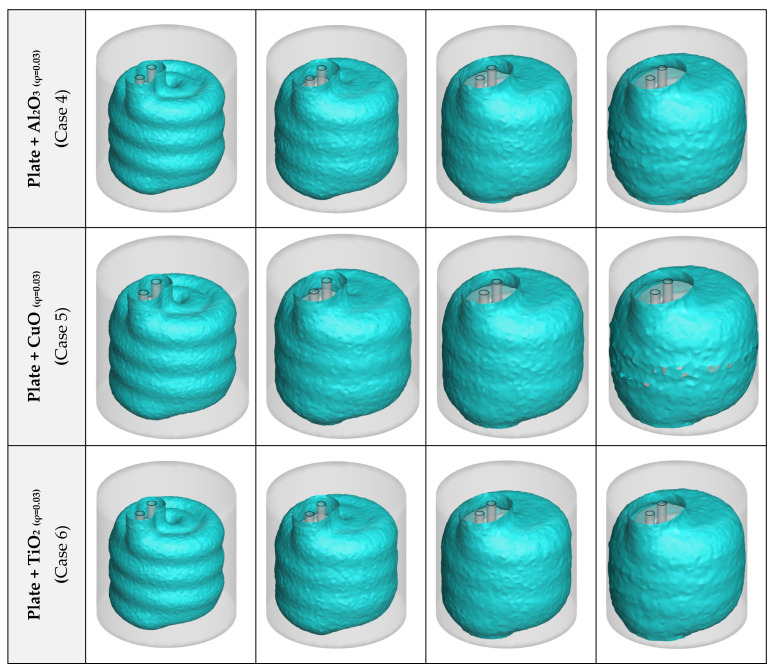
The 3D zero-liquid fraction iso-surfaces with insertion of plates and dispersion of various NP types in the PCM.

Figure 9 presents the 3D iso-surface images for the liquid fraction value of zero. These images illustrate the solidification front in the unit. As can be observed in this figure, the insertion of plates considerably increases the amount of formed ice over a specific amount of time. The dispersion of NPs leads to a similar result and advances the ice formation even further. However, as previously indicated, with different NPs the results are close and no considerable difference is found for the ice formation with various types of NPs. 

### 3.3. Nanoparticle Concentration

**Figure 10:**
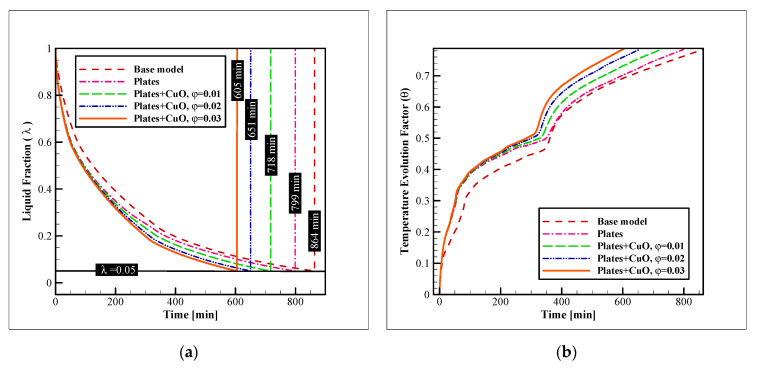
The impacts of the NP concentration and presence of plates on: (**a**) The liquid fraction reduction; and (**b**) TEF advancement.

This section investigates the impacts of dispersing various amounts of copper oxide NPs on the acceleration of the freezing process in the unit. Three different values of 0.01, 0.02, and 0.03 were tested as the CuO NP concentration (in volume). The results are presented in Figure 10. As can be observed, the NP concentration is considerably more effective in increasing the rate of the process than the NP material, as different values of NP concentration result in considerably different results for the complete freezing rate, while the different NP materials lead to quite similar results. The addition of the plate and copper oxide NPs with concentrations of 0.01, 0.02, and 0.03, improve the complete freezing rate by 16.89, 24.65, and 29.97%, respectively. The enhancement of the charging rate with the dispersion of the CuO NPs with these concentrations over the case with pure PCM and plates is 10.13, 18.52, and 24.28%, respectively. This shows that the addition of even 1% of CuO NPs (in volume) in the unit can be more effective than the insertion of the connecting plates. Comparing the enhancement obtained by the addition of NPs and plates reveal that while the dispersion of NPs creates an enhancement that constantly improves the freezing rate, the insertion of connecting plates is much more effective in the initial stages of the process, and when the progress advances and the ice layers cover the coils and plates, the case with plates loses its significant superiority over the base model. On the other hand, the enhancement acquired by the dispersion of NPs is not diminished over time and the cases with NPs keep their considerable superiority over the base model even in the final stages of the process. The same pattern exists in the TEF diagram and it is noticeable. 

**Figure 11:**
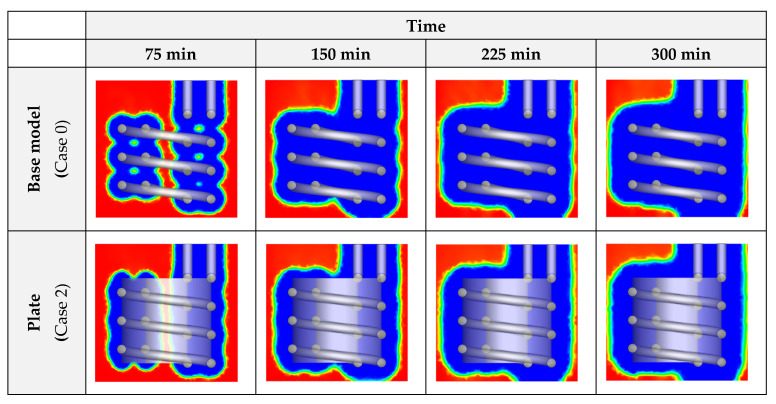

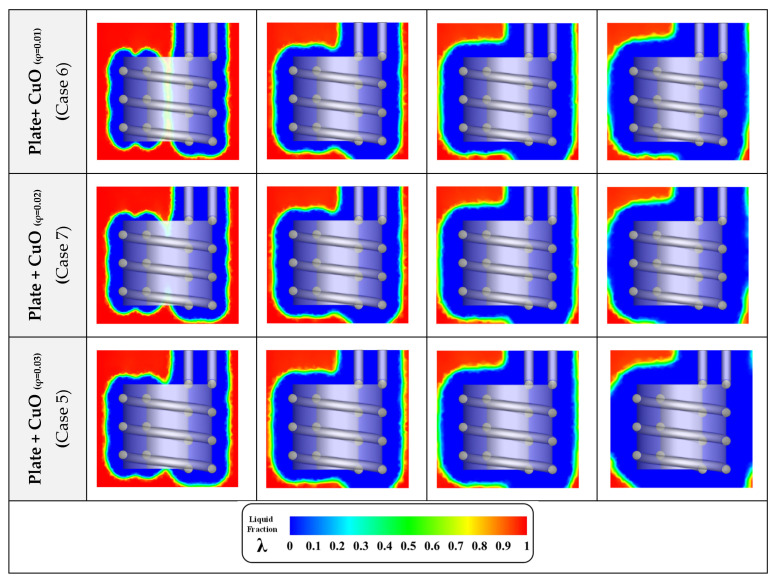
The changes in the liquid fraction contours of the unit with the insertion of plates and dispersion of various concentrations of CuO NPs in the PCM.

Figure 11 shows the liquid fraction contours with the insertion of plates and different percentages of CuO NP additives. As illustrated in the figures, at a certain time, the cases, in which CuO NPs with higher concentrations are dispersed, are more advanced in the progress of ice formation. Plates are also effective in this regard. Therefore, the use of these two improvement methods is a good way to significantly raise the response time in ice storage systems.

## 4. Conclusions and Recommendations

To solve the problem regarding the slow charging process of ice storage systems, the compound method of dispersing nanomaterials with high thermal conductivities in the PCM and the addition of connecting plates were studied using a series of complex 3D computational simulations. At first, three commercial materials were tested to select the best material for the HX tubes and fins, and then a compound enhancement technique was used to improve the charging rate. The utilized method included nano additives and connecting plates. Here are the highlights of this research:

- Considering the thermal performance with different HX materials, copper alloy C11000 presents the best results, however, the aluminum alloy 6061 has a similar thermal performance (just 5.44% lower). Selecting aluminum alloy for the HX is cheaper and makes the HX almost 70% lighter;
- The compound heat transfer augmentation of the ice storage systems with nano additives and connecting plates is significantly influential and can lead to enhancements up to 29.97% in the complete freezing time;
- The effects of NPs are higher than the plates, as the plates lose their influence after a certain amount of time since they are covered by ice, however, the NPs constantly improve the process until it ends;
- Among the tested NPs, CuO leads to the best results. However, the results with Al_2_O_3_ or even TiO_2_ are not much different;
- The concentration of NPs is considerably more effective on the freezing time than the material of the NPs.

Considering that this research was not focused on the application of nanomaterials on the refrigerant side, as a future study, the simultaneous use of NPs on both PCM and refrigerant sides of the system is recommended. Also, a two-phase model can be utilized and the results can be compared with the homogenous model that was employed for this research. Modeling the discharging process of this unit is also recommended for future studies.

## Figures and Tables

**Table 1:** Thermophysical properties of materials [6,21,22].

Property	PCM		HTF	HX			NPs		
	Water		Ethylene Glycol Solution	Aluminum Alloy 6061	Copper Alloy C 11000	Stainless Steel AISI 304	Al_2_O_3_	Cuo	TiO_2_
	Phase								
	Solid	Liquid							
ρ [kg·m^−3^]	917	999.8	8933	2700	8890	7900	3970	6510	4250
µ [Pa·s]	-	0.00162	-	-	-	-	-	-	-
c_p_ [J·kg^−1^·K^−1^]	2217	4180	385	963	385	477	765	540	686.2
k [W·m^−1^·K^−1^]	1.918	0.578	401	202.3	388	14.9	40	18	8.9538
h_sf_ [J·kg^−1^]	334,000		-	-	-	-	-	-	-
T_solidus_ [K]	273.15		-	-	-	-	-	-	-
T_liquidus_ [K]	273.15		-	-	-	-	-	-	-
β [K ^−1^]	-	−6.733353 × 10^−5^	-	-	-	-	0.85	0.85	0.9

**Table 2:** Cases defined for the examinations.

Studied Parameter	Case Number	HX Material	NP Type	NP Concentration
				φ [%]
**HX material**	1	Aluminum alloy 6061	-	0%
	2	Copper alloy C11000		
	3	Stainless steel AISI 304		
**NP type**	2	Copper alloy C11000	-	3%
	4		Al_2_O_3_	
	5		CuO	
	6		TiO_2_	
**NP concentration [%]**	2	Copper alloy C11000	CuO	0%
	7			1%
	8			2%
	5			3%

## Data Availability

The data presented in this study are available on reasonable request from the corresponding author.

## Nomenclature

$C_{mush}$	Mushy zone constant [kg·m^−3^·s^−1^]
$C_{P}$	Specific heat [J·kg^−1^ K^−1^]
d	The diameter [mm]
$\vec{g}$	Gravity [m·s^−2^]
h	Specific enthalpy [J·kg^−1^]
$h_{sf}$	Latent heat of fusion [J·kg^−1^]
k	Thermal conductivity [W·m^−1^·K^−1^]
m	Mass [kg]
Re	Reynolds Number
T	Temperature [K]
t	Time [s] or [hr]
V	Volume [m^3^]
$\vec{v}$	Velocity vector [m·s^−1^]
v	Velocity magnitude [m·s^−1^]
***Greek Symbols***	
β	Thermal expansion coefficient [K^−1^]
θ	Temperature evolution factor
λ	Liquid fraction
μ	Dynamic viscosity [Pa·s]
ρ	Density [kg·m^−3^]
φ	Nanoparticle volume fraction
***Subscripts***	
*0*	Reference
*b*	Base fluid
*cool*	Coolant
*f*	Fluid
*i*	In
*init*	Initial
*lat*	Latent
*liq*	Liquid
*liquidus*	Liquidus
*NEPCM*	Nano-enhanced PCM
*np*	Nanoparticles
